# Does axonopathy play a role in Parkinson's disease?

**DOI:** 10.1186/1750-1326-7-S1-L14

**Published:** 2012-02-07

**Authors:** Karen L O’Malley, Jo Ann Antenor-Dorsey, Jeong Sook Kim-Han

**Affiliations:** 1 Department of Anatomy and Neurobiology, Washington University in St. Louis, Saint Louis, MO, USA

## Background

Impaired axonal transport may play an early, pivotal role in a variety of neurodegenerative disorders, including Parkinson’s disease (PD). For example, postmortem studies on PD patients show widespread axonal pathology preceding the loss of cell bodies, animal models of PD-linked mutations such as the R1441G LRRK2 mutation exhibit decreased dopamine (DA) terminal fields together with increased dystrophic processes and abnormal axonal swellings, reduced axonal transport is also seen with α-synuclein mutants, and the PD-like toxin, MPP^+^, de-polymerizes microtubules leading to axon fragmentation as well as directly inhibiting axon transport in the isolated squid axoplasm. Thus, PD-linked environmental and genetic factors support the hypothesis that axon transport failure plays an early, critical role in this disorder.

## Methods

To discover how axon transport is disrupted in DA neurons, we established a culture system in which DA neurons are compartmentalized such that axons are on one side leaving cell bodies and dendrites on the other. When used with green fluorescent protein (GFP)-labeled DA neurons derived from genetically engineered mice, DA axons can be examined using live cell, real time imaging. Therefore we used cellular, optical, and pharmacological techniques to examine changes underlying toxin-mediated DA axonal impairment.

## Results

Our current studies show: (1) that the PD-mimetic MPP^+^ affects DA neuritic processes and microtubule tracks 12–18 h before cell bodies appear altered; (2) MPP^+^ halts mitochondrial trafficking in DA but not non-DA axons within 30 min; (3) remaining motile mitochondria exhibit decreased anterograde movement but increased retrograde trafficking; (4) MPP^+^ effects are specific for mitochondria, as synaptophysin-tagged vesicles and other detectable moving particles continue normal movements in either direction; (5) decreased mitochondrial trafficking is accompanied by a loss of ΔΨ_m_; (6) loss of mitochondrial movement is not associated with ATP loss but rather redox changes; (7) DA mitochondria are smaller and slower than non-DA organelles, suggesting cell type-specific differences exist for axonal mitochondria; and (8) the Wallerian degeneration slow fusion protein (*Wld^S^*) can rescue the effect of MPP^+^

## Conclusions

These studies confirm and dramatically extend the notion that axonal transport impairment plays a significant role in PD. Moreover, these findings underscore the necessity of developing therapeutics aimed at axons as well as cell bodies so as to preserve circuitry and function.

